# Thwarting predators? A three-dimensional perspective of morphological alterations in the freshwater crustacean *Daphnia*

**DOI:** 10.1371/journal.pone.0254263

**Published:** 2021-07-07

**Authors:** Martin Horstmann, Ralph Tollrian, Linda C. Weiss

**Affiliations:** Department of Animal Ecology, Evolution and Biodiversity, Ruhr-University Bochum, Bochum, Germany; University of Connecticut, UNITED STATES

## Abstract

Predation is a major selective agent, so that many taxa evolved phenotypically plastic defensive mechanisms. Among them are many species of the microcrustacean genus *Daphnia*, which respond to an increased predation risk by developing inducible morphological alterations. Some of these features are obvious and easily recognized, e.g., crests in *D*. *longicephala*, while others are rather hidden, such as the bulkier shape of *D*. *magna* induced by the presence of the tadpole shrimp *Triops*. In this study we investigated the extraordinary diversity of morphological adaptations in the presence of predators with different foraging strategies in six predator-prey systems. For the first time we were able to analyze the unexposed and predator-exposed morphs comprehensively using three-dimensional scanning and reconstruction. We show that morphological changes are manifold in appearance between species and predators, and go beyond what has been known from previous 2D analyses. This further demonstrates the enormous trait flexibility of *Daphnia*. Interestingly, we found that among this variety some species share morphological strategies to counter a predator, while others use a different strategy against the same predator. Based on these intra- and interspecific comparisons, we discuss the mechanisms by which the respective defense might operate. These data therefore contribute to a deeper understanding of the inducible defenses’ morphology as well as their diversified modes of operation in *Daphnia*, being a cornerstone for subsequent investigations, including the determination of costs associated with morphological change.

## Introduction

Predation is a major selective agent in ecosystems [1]. Therefore, many taxa evolved inducible morphological defenses to thwart predator attacks [2–4]. Microcrustaceans of the genus *Daphnia*, which are globally found in many freshwater ecosystems, have been intensely investigated and respond to an increased risk of predation conveyed by so-called kairomones and develop adaptive morphological features [5–10]. Some of these alterations are obvious and easily recognized, e.g. neckteeth in *D*. *pulex* [11, 12], crests in *D*. *longicephala* [13, 14], helmets in *D*. *cucullata* or *D*. *lumholtzi* [15, 16], while other shape changes are obscure such as the abandoned body symmetry in *D*. *barbata* [8, 17, 18]. Moreover, different types of hidden morphological changes have been described in various *Daphnia* species [19, 20]. Such adaptations are frequently correlated with an increased predation risk and apparently reduce it, however, in most species the morphological defenses’ modes of operation are only speculated if not left undetermined [e.g. 20–22].

Classically, these adaptations have been described using two-dimensional measurements between landmarks, often neglecting overall shape changes in all three dimensions due to technical limitations [e.g. 23, 24]. Only a few publications investigated changes in the lateral dimension. For example, the maximal lateral width was measured in *D*. *magna*, *D*. *pulex* and *D*. *longicephala*, i.e. the distance between the fornices tips respectively body torsion in *D*. *barbata* [22, 25]. Also, lateral width was found altered in specimens of *D*. *magna* exposed to *Triops* predation [26, 27]. Nevertheless, shape changes were only displayed based on individual measurements in this dimension. This provided only limited insight into the overall deformations underlying change expression and therefore functional assessments were limited. Even with the help of scanning electron microscopy, all three dimensions of the alterations can be depicted, but again only distance measurements were performed to describe potentially defensive morphologies, preventing a comprehensive analysis [17]. Also a PCA based approach with which the outline of shapes was compared is still limited to only 2 dimensions [28].

Since the functional mechanisms are unknown, it so far remains unclear which morphological alterations are necessary as defensive features, and which are by-products of actual defenses. Being able to produce detailed descriptions of these alterations is therefore pivotal for any kind of functional analysis. In order to bridge this gap concerning the three-dimensional morphology as well as the lack of mechanistical understanding, we employed our recently published workflow to capture all three dimensions of daphniids simultaneously [29] using geometric morphometric tools.

**Fig 1:**
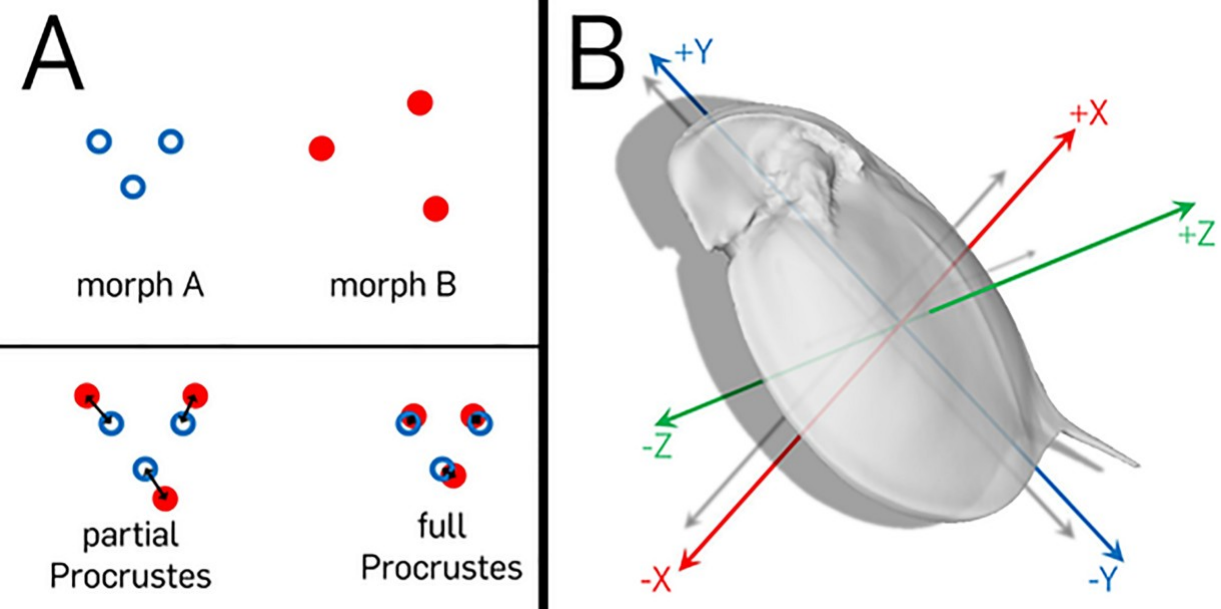
Procrustes distance and axes definition. (A) For Procrustes analysis, two landmark-sets, morph A and morph B, with an equal number of vertices (points) are required. Procrustes analysis allows to bring these two landmark sets in the best overlapping fit without changing their relative positions, using at least pan and rotation. This is called partial Procrustes fit and allows comparisons of form. If in addition to pan and rotation also scaling is adjusted, the result is a full Procrustes fit and allows shape analysis. Double-headed arrows give point-wise Procrustes distances, which are minimized during the analysis. The average Procrustes distance is the mean length of all arrows. Landmark-sets that differ only in scaling would overlap perfectly using full Procrustes, leading to a Procrustes distance of zero. (B) For the analysis of alterations, daphnids are inspected direction-wise: The dorso-ventral body axis is represented by the x-axis, with positive values towards the dorsal end. The longitudinal body axis is represented by the y-axis, with positive values towards the head. The lateral body axis is represented by the z-axis, with positive values out of the drawing plane, towards the viewer.

In morphological comparisons using this powerful toolbox, landmarks are fitted with a full Procrustes analysis [30, 31]. This removes differences in position, rotation and size, and allows the comparison of shape, following the classical definition of shape as a geometric object without information on the mentioned geometric parameters [31–33]. When only position and rotation are removed from landmark-sets by a partial Procrustes analysis, they still include differences in shape but also size alterations, which in sum we define as form alterations (Fig 1A). This procedure allows to unveil the locations of morphological alteration in detail.

As an objective measure of shape similarity, we additionally determined average Procrustes distances. These are calculated based on the square root of the sum of the squared distances between respective body regions of each model fitted by Procrustes analyses, divided by the number of vertices, i.e. points on the surface of that model [30, 31, 34]. Since Procrustes is per definition unbiased by the user, the determined distance gives an objective value of alteration between two models. Using this measure, we compare the species three-dimensionally which is condensed in two numbers, the average full- and partial Procrustes distance. Large numbers of the full Procrustes distance represent strong alteration in shape between two models. By also calculating the partial Procrustes distance, we can determine whether the form dissimilarity is mainly because of size or due to an overall alteration of shape.

With the help of this approach, we aimed to quantify an excerpt of the different morphological alterations shown by various *Daphnia* species in order to understand functional principles and to enable downstream analyses, e.g. an assessment of costs associated with the formation of morphological alteration. We investigated five *Daphnia* species that were exposed to their co-existing predators. We exposed *D*. *magna* to the notostracan *Triops*, *D*. *barbata* to *Triops* and the heteropteran backswimmer *Notonecta*, *D*. *longicephala* to *Notonecta*, *D*. *pulex* to its respective predator, the dipteran phantom midge larvae *Chaoborus* and *D*. *lumholtzi* to fish (for their evolutionary origins see [35]).

With these data, we are able to confirm or refute hypotheses on the functional strategies of potential morphological defenses and enable new insights to deepen our understanding of the mechanisms behind.

## Methods

### Daphnia culture

*Daphnia* reproduce by cyclical parthenogenesis, so that experiments can be done on multiple individuals of the same genotype (i.e. clonal isolates). Clones of the investigated *Daphnia* species (*D*. *magna*, *D*. *barbata*, *D*. *longicephala*, *D*. *pulex*, *D*. *lumholtzi*) originated from various locations (Table 1). All animals were cultured in our institute for several generations under laboratory conditions (16:8-hour day-night cycle, 20°C +/- 1°C). Animals were reared in 1 L glass beakers (J. Weck GmbH und Co. KG, Wehr-Öflingen, Germany) containing charcoal filtered tap water and fed *ad libitum* with the green algae *Acutodesmus obliquus* every other day. Culture beakers and beakers of the experiments were cleaned in the same regular cycle. Water was exchanged weekly.

**Table 1:** Investigated *Daphnia* species.

species (clone)	body size (predator-exposed)	origin	predator (in experiment)	partial Procrustes distance	full Procrustes distance
*D*. *magna* (K34Q)	~2500 (adult)	Ismaninger fish ponds near Munich, Germany	*Triops* sp.	80.005 (3%)	27.529 (1%)
*D*. *barbata* (BO)	~1500 (adult)	Ethiopia, provided by C. Laforsch, originally provided by J. Mergeay	*Triops* sp.	26.259 (2%)	26.722 (2%)
*D*. *barbata* (BO)	~1500 (adult)		*Notonecta* sp.	40.602 (3%)	26.216 (2%)
*D*. *longicephala* (LP1)	~3000 (adult)	ephippia collected at Lara-Pond, Australia	*Notonecta* sp.	261.018 (9%)	139.090 (5%)
*D*. *pulex* (R9)	~700 (2^nd^ juvenile)	Canada, provided by L. Weider	*Chaoborus* sp.	10.538 (2%)	10.539 (2%)
*D*. *lumholtzi* (LA2)	~1500 (adult)	Lousiana (US), provided by L. Weider	*Gasterosteus aculeatus*	40.626 (3%)	40.929 (3%)

Body sizes at investigation, predator-exposed instars, origin and average partial as well as full Procrustes distances within species. All values given in micrometer. In addition, we give the Procrustes distances in percent of the body size of predator-exposed animals.

### Predator culture

*Chaoborus obscuripes* larvae and *Notonecta glauca* were caught in the ponds of the botanical garden of the Ruhr-University Bochum. *Chaoborus* larvae of the 4^th^ juvenile instar were kept in 1.5 L glass jars at 4° C in charcoal-filtered tap water. Adult *N*. *glauca* were kept at 15° C in 1 L glass beakers (J. Weck GmbH und Co. KG, Wehr-Öflingen, Germany) and fed with five adult daphniids and three defrosted chironomid larvae daily. *Triops* sp. were hatched from sand in 1.5 L tanks and each was fed daily with five adult daphniids, three defrosted chironomid larvae and one pellet of commercial fish food (Multisticks, Tetra GmbH, Melle, Germany). All *Triops* used for experiments had a body length larger than 1 cm. *Gasterosteus aculeatus* (obtained from Zoo Zajac GmbH, Duisburg, Germany) with a length of 5–7 cm were fed frozen chironomid larvae and a range of *Daphnia* sp. in the culture aquaria every other day.

### General induction procedures

Morphological alterations in the investigated *Daphnia* species were induced by exposing them to the predators, respectively remains of consumed conspecifics. We avoided predation on experimental organisms by keeping predators in net cages made of acrylic cylinders of 12 cm height and 7 cm diameter, lined with 125 μm gauze (Hydrobios, Germany), still allowing kairomone exposure. Depending on the predators, we fed the predators with conspecific daphniids during the exposures, as mentioned in the explicit descriptions below. In order to keep the concentration of predator kairomones stable we did not exchange water. Controls were handled similarly, but without the predators. To reveal maximal defense expression for the functional analysis, in all species, we selected strongly induced individuals. Morphological changes were assessed when *Daphnia* deposited eggs in the brood pouch, except for *D*. *pulex*, where we investigated the second juvenile instar, as this is the instar of strongest morphological alteration. For all predator treatments and the respective controls we used 7–16 individuals, which were taken from three replicates per species.

### Predator exposures

#### *Triops*-exposed *Daphnia magna*

We induced morphological alterations by exposing 10 *D*. *magna* neonates (first instar juveniles) to chemical cues from one *Triops* sp. We raised *D*. *magna* until animals deposited eggs in the brood pouch. Once these offspring were released, adult females were removed, while the neonates remained in the glass jars. This procedure was repeated for three generations [19, 26], always keeping the number of animals per 1.5 L jar at a maximum of 10 by randomly removing surplus neonates.

#### *Triops-* and *Notonecta*-exposed *Daphnia barbata*

*D*. *barbata* was shown to develop contrasting features against *Notonecta* and *Triops* [17, 21]. We therefore exposed 20 *D*. *barbata* from the 5^th^ embryonic stage [36] onwards to 1 individual of both predators independently, again using net cages placed in 1.5 L beakers. *Notonecta* and *Triops* were fed as described above in predator culture.

#### *Notonecta*-exposed *Daphnia longicephala*

*D*. *longicephala* was exposed in groups of 10 individuals to predator cues of the heteropteran backswimmer *Notonecta glauca* from the first juvenile instar onwards using 1 L glass beakers. We used one *Notonecta* per net cage fed with 5 adult *D*. *longicephala* initially and three *D*. *longicephala* on subsequent days.

#### *Chaoborus*-exposed *Daphnia pulex*

Inductions were started with 5 female *D*. *pulex* carrying embryos in the last embryonic stage, showing black eyespots [36], placed in 1 L glass beakers. For predator exposure, we placed 10 *Chaoborus* larvae and once 100 juvenile *D*. *pulex* as food source for the predator in the net cage.

#### *Gasterosteus*-exposed *Daphnia lumholtzi*

*D*. *lumholtzi* were exposed to cues from the three-spined stickleback *Gasterosteus aculeatus* from the 5^th^ embryonic stage onwards in groups of 20 individuals in a 1 L glass beaker. For that, one stickleback was placed in a net cage positioned inside the beaker for 8 hours per day, without feeding during that time.

### Animal fixation, staining and scanning

After reaching the respective instar, animals were fixed, stained and processed as described previously [29]. In short, we fixed the animals with 4% formaldehyde (J.T. Baker, Germany) diluted in phosphate buffered saline (PBS; pH 7.4, 0.1 M), stained them with 0.1% Congo red (Carl Roth GmbH + Co. KG, Karlsruhe, Germany) and scanned them with confocal microscopes (Leica TCS SP5II (Leica Microsystems, Wetzlar, Germany), Zeiss LSM 710 (Carl Zeiss Ltd., Cambridge, UK) and Nikon A1R (Nikon Instruments Europe B.V., Amsterdam, Netherlands)). We used the automatic image stitching when necessary for large specimens.

### Modelling

Three-dimensional meshes were extracted from the stacks using MorphoGraphX [37]. After projecting a grid onto the meshes using Blender (Blender, version 2.73, Blender Foundation, Blender Institute Amsterdam, https://www.blender.org/, 2015), the resulting individual models consisting of comparable vertices, which means points on the surface, were averaged to a single model per treatment, using an automated Matlab Script [29] (Matlab R2014b, The Mathworks Inc., Natick, MA, 2015). Here, Procrustes analysis was used to superimpose the models in a best overlapping fit (Fig 1). With this script, we also determined displacement vectors, i.e. vectors between respective vertices in both morphs’ models. We projected their length with color gradients on the 3D surface reconstructions in summation (subfigures C) or directions-wise (subfigures D, F, H). Also, Wilcoxon-tests were calculated for the position of vertices in all three dimensions of space between specimens of the unexposed and predator-exposed treatment. To correct for error based on multiple testing with the False Discovery Rate (FDR)-analysis, we calculated q-values, which give a value that allows to evaluate the trustworthiness of each derived p-value [38]. These q-values are based on the FDR, which is estimated from the distribution of p-values of the conducted Wilcoxon tests (subfigures E, G, I). All original datasets as well as the resulting Matlab script and workspace files are available via figshare (https://doi.org/10.6084/m9.figshare.14269625.v1, https://doi.org/10.6084/m9.figshare.14269409.v1.).

Using this 3D morphometric approach, we obtained a comprehensive overview of morphological alterations in the investigated *Daphnia* species. We describe the significant changes in adaptive morphological features in detail using the nomenclature of body parts listed in S1 Fig. In the following, shifts are described with ‘strong tendency’ when the Wilcoxon-tests are represented with p-values smaller than 0.05 and regarded significant when respective q-values calculated with the FDR-analysis are smaller than 0.01. To display directional changes, we show point translocation in a direction-specific manner across the x-, y-, and z-axis.

### Comparison of intra- and interspecies differences with Procrustes distances

In addition, we calculated Procrustes distances (in micrometer), which represent the sum of the distances of respective vertices after a Procrustes analysis using Matlab (Matlab R2014b, The Mathworks Inc., Natick, MA, 2015). While partial Procrustes allows comparisons of form, including size effects, full Procrustes removes rotation, translation and scaling from shapes (Fig 1A). For further information refer to [29].The Procrustes distances were calculated between the two treatments of each species to determine the alterations’ magnitude (Table 1). Furthermore, we determined the Procrustes-distances between all models of predator-exposed specimens, which enables to evaluate, how strong shape and form differ between these morphs (Table 2). For the latter, we again adjusted and projected a grid suitable to fit onto all models. This grid contained 432,321 vertices, from these, 384,019 vertices could be projected due to the irregular shape and were accordingly used for the calculation of the interspecies-Procrustes-distance. For the partial Procrustes distances, the size adjustment of the models before the calculation was excluded in the analyses via Matlab, for the determination of full Procrustes distances it was included. As Procrustes distances are dependent on the morphological distance between two morphs, but also influenced by the number of vertices used for the comparison, we divided all Procrustes distances by the number of vertices on which they are based, giving average partial and full Procrustes distance, which represent an average offset between the vertices of the point clouds of both morphs (Tables 1 and 2). Despite the magnitude of alterations which can be examined thoroughly, the actual morphological change of these alterations is not captured. Accordingly, the same numbers do not necessarily describe the same morphologies.

**Table 2:** Average partial and full Procrustes distances between species.

	Partial P	*D*. *magna (Triops)*	*D*. *barbata (Triops)*	*D*. *barbata (Notonecta)*	*D*. *longicephala (Notonecta)*	*D*. *pulex (Chaoborus)*
	Full F					
*D*. *magna (Triops)*	P	x	x	x	x	x
	F					
*D*. *barbata (Triops)*	P	1542.5	x	x	x	x
	F	168.2				
*D*. *barbata (Notonecta)*	P	1698.9	533.3	x	x	x
	F	192.7	34.7			
*D*. *longicephala (Notonecta)*	P	2161.7	744.5	587.3	x	x
	F	258.1	185.0	174.0		
*D*. *pulex (Chaoborus)*	P	1447.2	331.9	403.1	786.7	x
	F	34.9	45.1	56.0	487.6	
*D*. *lumholtzi (Gasterosteus)*	P	1468.1	532.4	704.8	921.1	455.8
	F	83.0	79.1	60.8	94.5	96.1

P gives the partial Procrustes distance, F the full Procrustes distance. All distances are given in micrometer.

### Ethics approval and consent to participate

All animals used in this study were maintained according to animal welfare regulations.

## Results

We here describe the relevant, significant alterations between the predator-unexposed and -exposed morphs. For a detailed description of all changes, please see (S1–S6 Text in S1 File).

### *Daphnia magna*: *Triops-*induced morphological alterations

**Fig 2:**
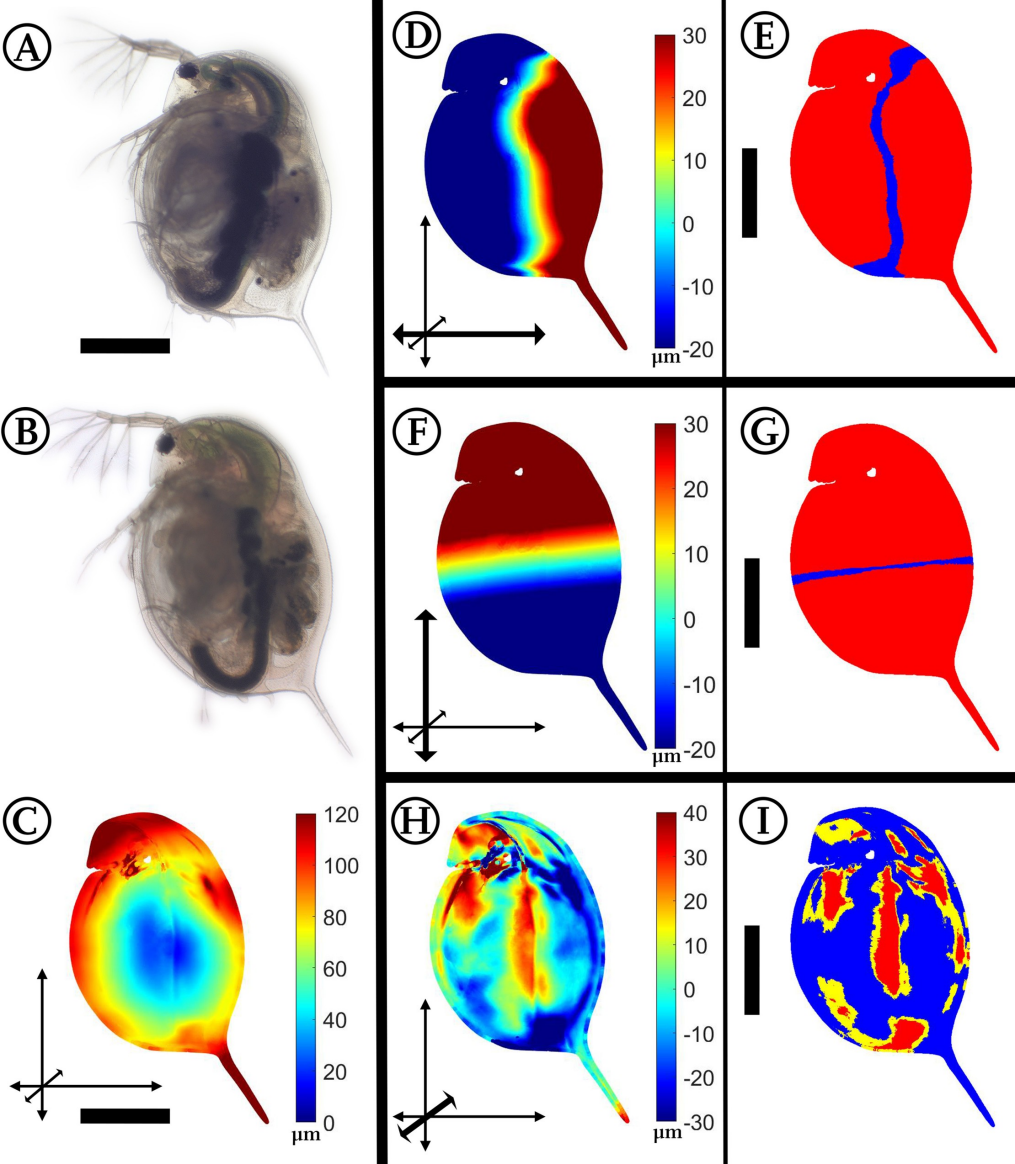
Analysis of *Triops*-exposed *Daphnia magna*. (A) *D*. *magna*, unexposed. (B) *D*. *magna*, *Triops*-exposed. (C) Overall deformation plot showing the sites of cumulated alteration with blue giving zero and red maximal alteration. (D, F, H) Vertex displacements along the coordinate axes (x, y, z respectively). Shades of blue colours represent alterations in negative direction, while shades of red display shifts in positive direction on the respective axis. Note the altered colour scale. The coordinate system gives the relevant axis marked in bold (see also Fig 1B). (E, G, I) p-values lower than 0.05 displayed in yellow, regions additionally supported by q-values lower than 0.01 based on the FDR-analysis are coloured red. n_unexposed_ = 10, n_predator-exposed_ = 9. All scale bars: 1 mm.

*Triops*-exposed (n = 10) compared to -unexposed (n = 9) *D*. *magna* (Fig 2) have maximal alterations at the body margins as well as at the tail spine. In the lateral direction, we observed an increased body width at a rim on the carapace, as well as on the fornices in *Triops*-exposed animals. At the same time, the posterior ventral margin and the dorsal margin are slightly slimmer in predator-exposed *D*. *magna*. Summing up all alterations, *D*. *magna* confronted with the predator shows an overall increase in body dimensions.

### *Daphnia barbata*: *Triops*-induced morphological alterations

**Fig 3:**
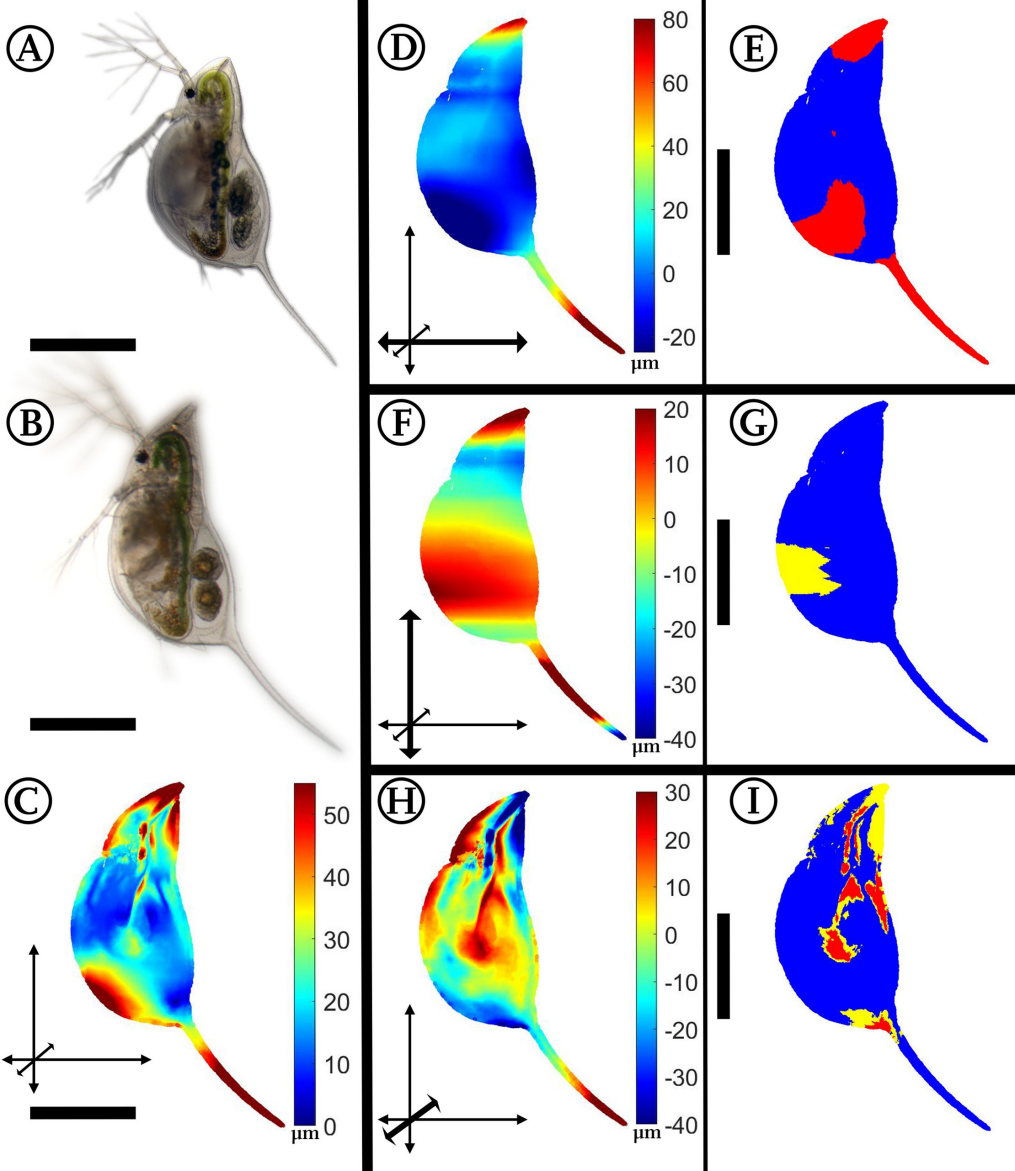
Analysis of *Triops*-exposed *Daphnia barbata*. (A) *D*. *barbata*, unexposed. (B) *D*. *barbata*, *Triops*-exposed. (C) Overall deformation plot showing the sites of cumulated alteration with blue giving zero and red maximal alteration. (D, F, H) Vertex displacements along the coordinate axes (x, y, z respectively). Shades of blue colours represent alterations in negative direction, while shades of red display shifts in positive direction on the respective axis. Note the altered colour scale. The coordinate system gives the relevant axis marked in bold (see also Fig 1B). (E, G, I) p-values lower than 0.05 displayed in yellow, regions additionally supported by q-values lower than 0.05 based on the FDR-analysis are coloured red. n_unexposed_ = 7, n_predator-exposed_ = 7. All scale bars: 600 μm.

Maximal changes of form in *D*. *barbata* exposed to *Triops* (n = 7) in comparison to unexposed individuals (n = 7) occur at the tip of the tail spine and at the head (Fig 3). In the lateral dimension, the shape is asymmetric. While the head is shifted to the animal’s right, the tail spine is displaced to the left. Additionally, the carapace fold is more pronounced in the lateral dimension in predator-exposed than in unexposed *D*. *barbata*. Not only this fold, but also the whole carapace is expanded by 20 μm.

### *Daphnia barbata*: *Notonecta*-induced morphological alterations

**Fig 4:**
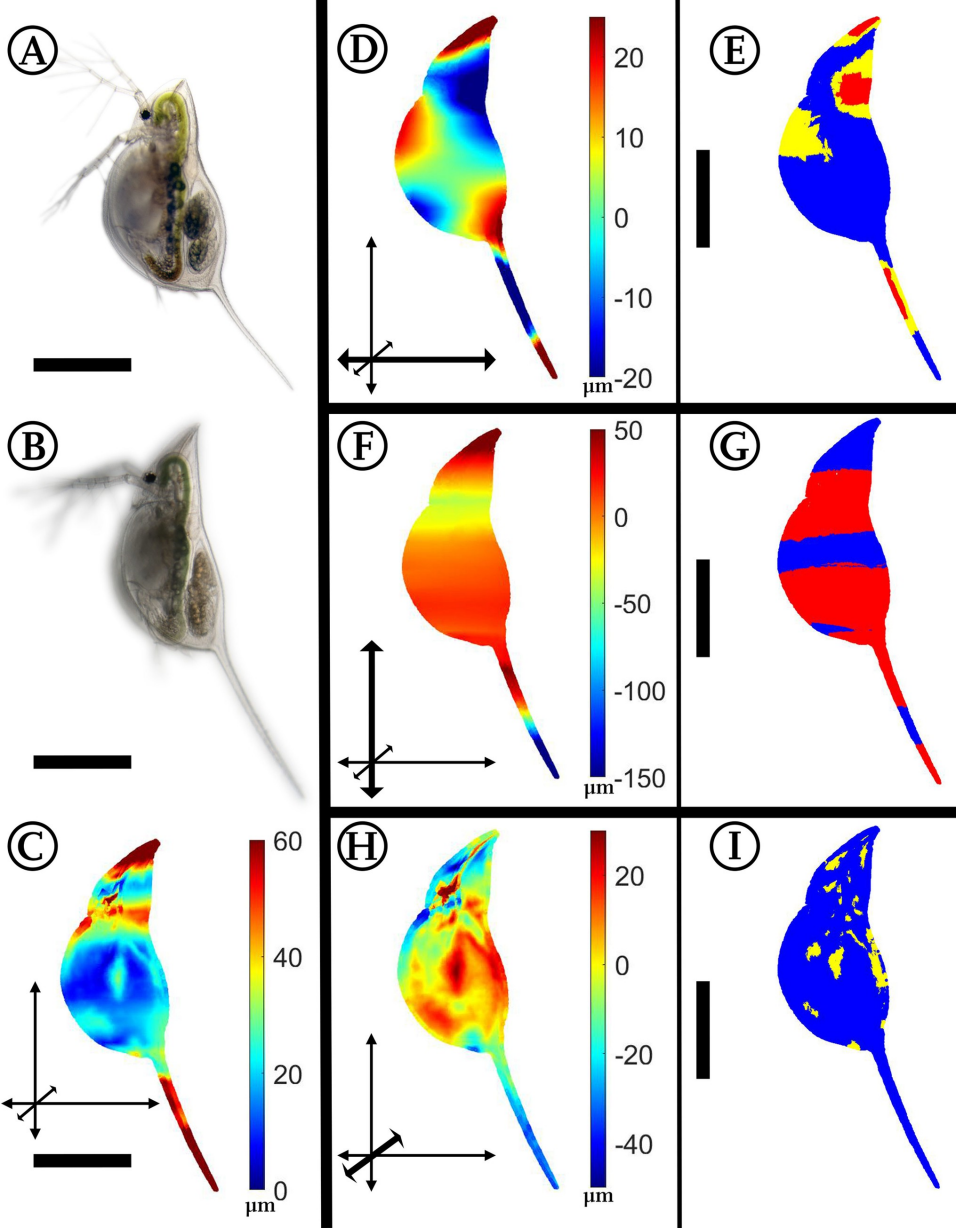
Analysis of *Notonecta*-exposed *Daphnia barbata*. (A) *D*. *barbata*, unexposed. (B) *D*. *barbata*, *Notonecta*-exposed. (C) Overall deformation plot showing the sites of cumulated alteration with blue giving zero and red maximal alteration. (D, F, H) Vertex displacements along the coordinate axes (x, y, z respectively). Shades of blue colours represent alterations in negative direction, while shades of red display shifts in positive direction on the respective axis. Note the altered colour scale. The coordinate system gives the relevant axis marked in bold (see also Fig 1B). (E, G, I) p-values lower than 0.05 displayed in yellow, regions additionally supported by q-values lower than 0.05 based on the FDR-analysis are coloured red. n_unexposed_ = 7, n_predator-exposed_ = 8. All scale bars: 600 μm.

Maximal changes between unexposed (n = 7) and predator-exposed animals (n = 8) occur at the tail spine and at the tip of the helmet, which are more pointed, the rostrum, and the region between the head capsule and the carapace (Fig 4). In the lateral dimension, the width of the fold on the head and the tip of the helmet is increased, while the carapace is not or just slightly thickened. Attenuation of lateral width is found on the tail spine, the ventral margin, and the ventral parts of the head capsule.

### *Daphnia longicephala*: *Notonecta*-induced morphological alterations

**Fig 5:**
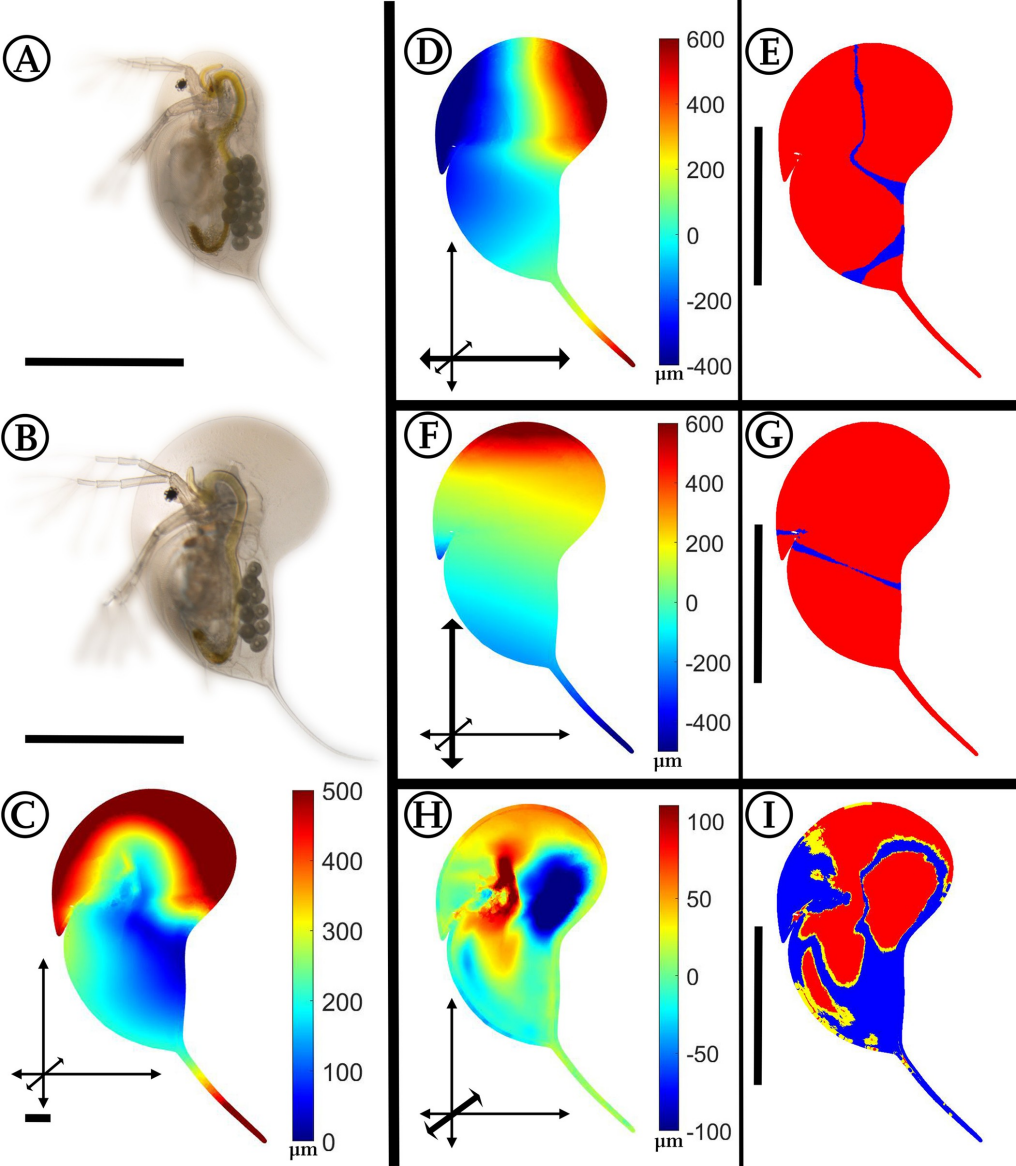
Analysis of *Notonecta*-exposed *Daphnia longicephala*. (A) *D*. *longicephala*, unexposed. (B) *D*. *longicephala*, *Notonecta*-exposed. (C) Overall deformation plot showing the sites of cumulated alteration with blue giving zero and red maximal alteration. (D, F, H) Vertex displacements along the coordinate axes (x, y, z respectively). Shades of blue colours represent alterations in negative direction, while shades of red display shifts in positive direction on the respective axis. Note the altered colour scale. The coordinate system gives the relevant axis marked in bold (see also Fig 1B). (E, G, I) p-values lower than 0.05 displayed in yellow, regions additionally supported by q-values lower than 0.05 based on the FDR-analysis are coloured red. n_unexposed_ = 14, n_predator-exposed_ = 16. All scale bars: 2 mm.

In comparison to the unexposed morph (n = 14), the predator-exposed morph of *D*. *longicephala* (n = 16) is characterized by a pronounced crest (Fig 5). The crest and the tail spine show the highest degree of overall deformation. In the lateral direction the folds on the head capsule are increased in thickness. Apart from these very strong alterations, the dorsal and ventral margins are decreased in thickness.

### *Daphnia pulex*: *Chaoborus*-induced morphological alterations

**Fig 6:**
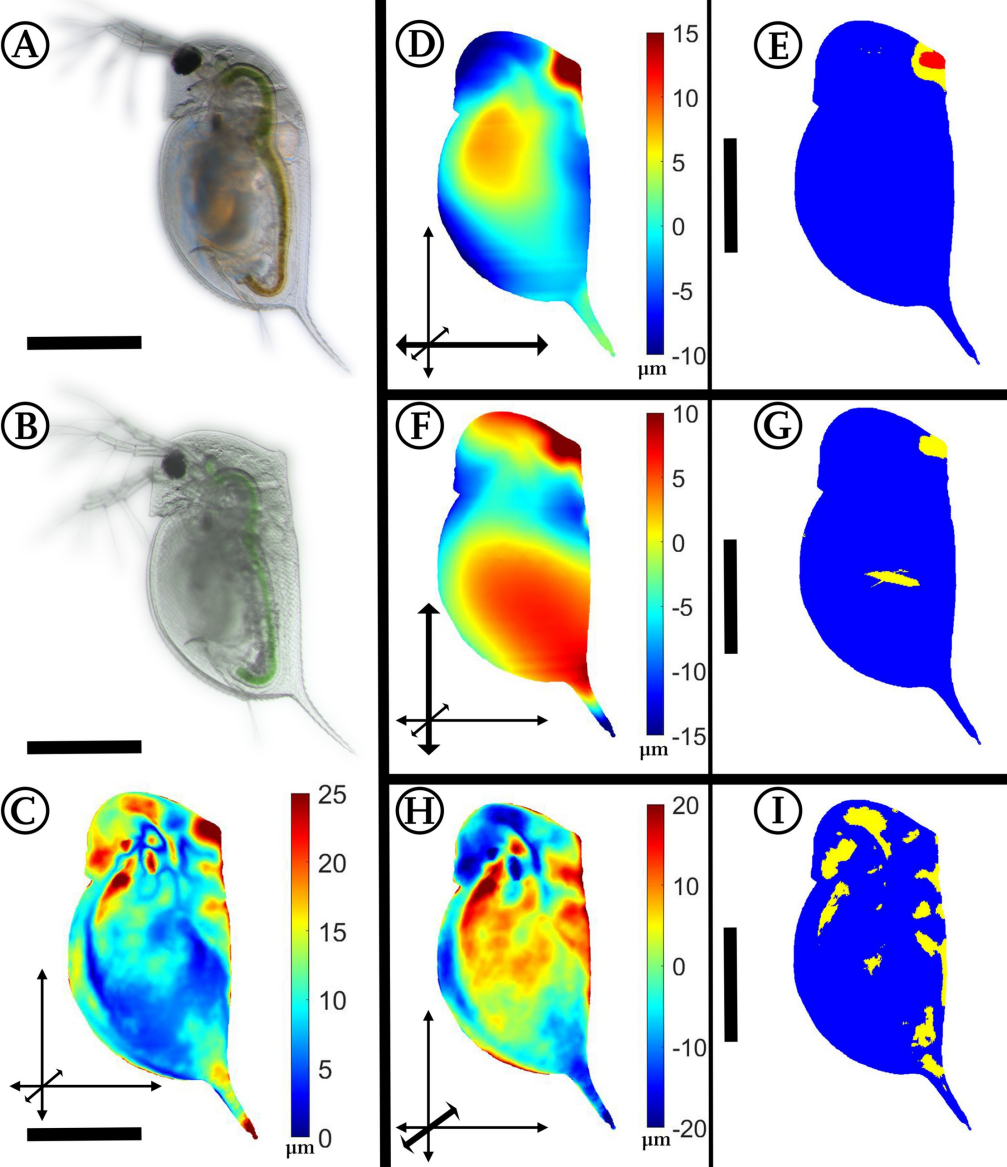
Analysis of *Chaoborus*-exposed *Daphnia pulex*. (A) *D*. *pulex*, unexposed. (B) *D*. *pulex*, *Chaoborus*-exposed. (C) Overall deformation plot showing the sites of cumulated alteration with blue giving zero and red maximal alteration. (D, F, H) Vertex displacements along the coordinate axes (x, y, z respectively). Shades of blue colours represent alterations in negative direction, while shades of red display shifts in positive direction on the respective axis. Note the altered colour scale. The coordinate system gives the relevant axis marked in bold (see also Fig 1B). (E, G, I) p-values lower than 0.05 displayed in yellow, regions additionally supported by q-values lower than 0.05 based on the FDR-analysis are coloured red. n_unexposed_ = 7, n_predator-exposed_ = 8. All scale bars: 400 μm.

In *Chaoborus*-exposed (n = 8) in comparison to unexposed *D*. *pulex* (n = 7), strongest shifts in morphology occur in the neckteeth region (Fig 6). This displacement is mainly due to a shift in dorsal direction. In the lateral dimension we observe a decrease in the head’s thickness. Accordingly, also the fornix is attenuated. The heart region is characterized by an alternating pattern of positive and negative displacement in this direction.

### *Daphnia lumholtzi*: *Gasterosteus*-induced morphological alterations

**Fig 7:**
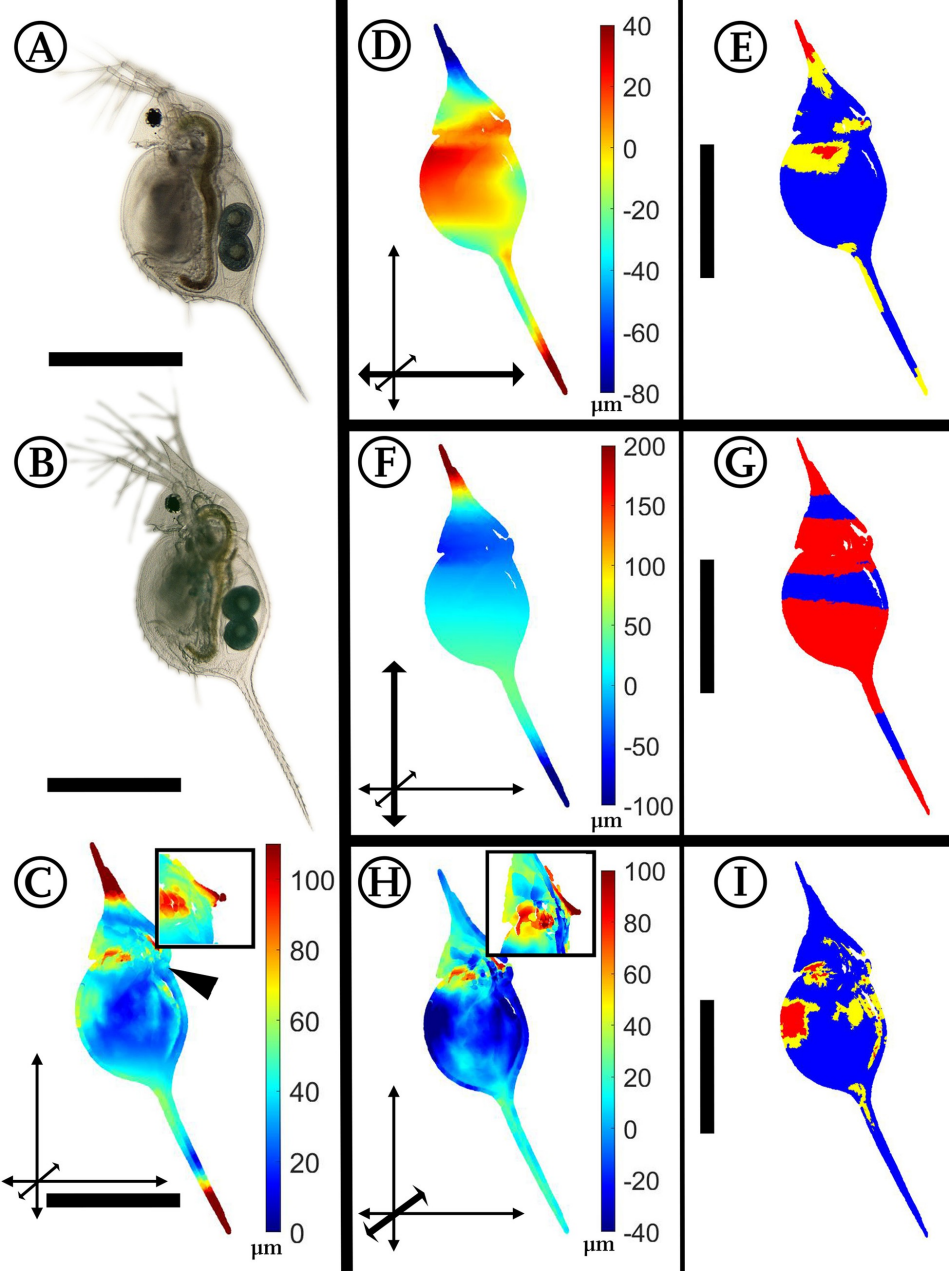
Analysis of *Gasterosteus*-exposed *Daphnia lumholtzi*. (A) *D*. *lumholtzi*, unexposed. (B) *D*. *lumholtzi*, *Gasterosteus*-exposed. (C) Overall deformation plot showing the sites of cumulated alteration with blue giving zero and red maximal alteration. The insert shows a detail of the head and especially the region of the fornices. (D, F, H) Vertex displacements along the coordinate axes (x, y, z respectively). Shades of blue colours represent alterations in negative direction, while shades of red display shifts in positive direction on the respective axis. Note the altered colour scale. The coordinate system gives the relevant axis marked in bold (see also Fig 1B). Subfigure H additionally gives an insert with a detail of the laterally strongly extended fornices. (E, G, I) p-values lower than 0.05 displayed in yellow, regions additionally supported by q-values lower than 0.05 based on the FDR-analysis are coloured red. n_unexposed_ = 10, n_predator-exposed_ = 8. All scale bars: 400 μm. ©Fig 7A and 7B: Graeve/Janssen.

Obvious alterations of *D*. *lumholtzi* in *Gasterosteus* presence (n = 8) compared to unexposed animals (n = 10) are an elongated tail spine and a pointed helmet (Fig 7). The strongest alterations are observable at the tip of the tail spine and the tip of the pointed head capsule. Strong lateral deformations occur at the fornices. These laterally extruded body appendages of the head capsule enhance the absolute width of exposed animals significantly by 200 μm through being more pointed (Fig 7C and 7H insert), while some areas appear thinned in the lateral dimension in predator-exposed animals, e.g. regions at the dorsal and ventral margins of the carapace.

### Procrustes distances

Smallest Procrustes distances were calculated for *D*. *pulex* and largest distances for *D*. *longicephala* (Table 1). Marginal differences between partial and full Procrustes distance were found between *D*. *barbata* in the presence of *Triops*, *D*. *lumholtzi* and *D*. *pulex*. Most species showed partial Procrustes distances in the range of 1–3% and full Procrustes distances of 1–2% of their body size. The largest difference between partial and full Procrustes distances was measured in *D*. *longicephala*. Here, we also found the largest average full Procrustes distance, as well as the largest Procrustes distances in relation to body size (Table 1).

In addition, the partial and full Procrustes distances between the predator-exposed morphs of all species were calculated (Table 2). To normalize these values, we again used the average distances over all points of a model. These values were generally larger than the values found for the intraspecific Procrustes distance. Typical values for the partial analysis comprising only smaller species range around 500 μm, if larger species are part of the comparison, we find values up to 2161 μm. For the full Procrustes distance, typical values are smaller, the larger portion of comparisons does not exceed 200 μm. In the case of comparisons including *D*. *lumholtzi*, often values of less than 100 μm resulted for the full Procrustes analyses. Even in the comparison with *D*. *longicephala*, the full Procrustes value is smaller than 100 μm, while in all other species we obtained values ranging from 174–488 μm. The predator-exposed morph of *D*. *lumholtzi* is due to the pointed head more similar to *D*. *longicephala* than all other species.

## Discussion

Based on the three-dimensional models of selected predator-unexposed and -exposed morphs of different *Daphnia* species we analyzed the intra- as well as interspecific alteration occurring in predator presence. We especially emphasized on the overlapping predator regimes to find evidence for the relevant morphological features in the overall alteration.

### Comparison of morphological alterations

At a first glance, morphological changes in the different predator-exposed animals appear specific to the predator. However, analyzing the three-dimensional alterations in more detail allows to detect adaptive patterns overarching several species.

#### *Triops*-induced morphologies

We recently described explicit morphological changes in shape and form of *D*. *magna* in the presence of *Triops* sp. [29]. In the current study we confirm our previous results and here also found that *D*. *magna* reacts by increasing its overall body size. We find that the body length and dorso-ventral width of the animals was not only locally increased as Rabus et al. [21, 26] described, but extended in the overall outline. In addition, we also describe that the fornix’ width is significantly increased.

In comparison to *D*. *magna*, *D*. *barbata* expresses different morphological adaptations in presence of *Triops*. In *Triops*-exposed *D*. *barbata* we see elongated and twisted helmets and tail spines. The animals’ helmets are shifted to the right in *Triops*-exposed specimens, while the tail spines bend to the left body half, causing a significant asymmetry, which was also described by Herzog et al. [17, 22]. In addition to this observation, we find in our 3D analysis that the ventral margin is especially convex at the posterior end, while the tail spine and helmet are bent backwards. Overall, these adaptations follow the form of a sickle.

The described shifts of *D*. *magna* and *D*. *barbata* in the presence of *Triops* are similar in the overall body outline which becomes more circular. While *D*. *magna* simply grows bulkier, which is thought to hamper *Triops* predation and to influence the prey choice of *Triops* [26, 39–41], *D*. *barbata* bends its body, forming a sickle. As this circular arc is approximately closed under strong expression, it may resemble the ‘filled’ circular outline of *D*. *magna*. Both shapes may help to reduce the contact zone between the *Triops*’ gnathobases forming the food groove [17]. It is possible that the asymmetric shape also helps *D*. *barbata* to be swirled out of the food groove. Similarly, the asymmetry could hamper an immediate firm grasp of the prey during free swimming. *Triops* also hunt by trapping the prey between its body and the ground [42]. This may be impeded through the circular body outline, and thereby increase the *daphniids* survival chances. Furthermore, the increase in the lateral width may help both species to outgrow the mouth opening of this gape limited predator.

#### *Notonecta*-induced morphologies

In contrast to *Triops*-exposed *D*. *barbata*, in *Notonecta*-exposed *D*. *barbata* we found especially pronounced helmets and tail spines, which were previously reported by [17, 18, 22]. Furthermore, in the area around the center of the dorsal margin and centrally on the carapace we observe an increased lateral width. It has previously been discussed that animals exposed to *Notonecta* are well-protected also against *Triops*. This form change appears effective in multiple environments [22].

Forming a pronounced and straight helmet in combination with a pointed body shape could disable predator handling. Furthermore, the dorsal body margin could be the region where *Notonecta* pierces its proboscis through the carapace. Therefore, an increased lateral thickness may serve as a regional fortification, based not on the material properties, but its form, as this could aid a diversion of the proboscis away from this vulnerable region. A more rounded shape furthermore can help to distribute forces more evenly and therefore improves stability [43]. This mechanism is probably similar in the morphological change of *D*. *barbata* in the presence of *Triops*.

We speculate that the reduced lateral width at e.g., the ventral margin, which is often accompanied by a longer narrowed channel into the ventral gap, contributes to a defense by hampering the piercing into this gap, as it is harder for the predator to find. Furthermore, such narrowing may also help to divert the proboscis of the predator during piercing. Especially as Daphniidae are able to actively narrow their ventral cleft [44], this special shape appears as a useful support to avoid punctures at this position.

*Notonecta* also elicits a morphological response in *D*. *longicephala* [13, 45–47], which appears totally different from *Notonecta*-exposed *D*. *barbata* at a first glance. Considering the overall shape of this species, we find an extension of the body in the sagittal plane. In principle, the overall shape is comparable with that of predator-exposed *D*. *magna*, as the three-dimensional shape with a central bulge and a thinned body periphery occurs in both species.

In the lateral dimension, the extensions are very thin. The head capsule’s lateral width is increased centrally, mainly because of the fornices forming a rim dorsal at the antennas’ base. We hypothesize that these changes function to increase the structural stability of the head capsule and carapace, as suggested previously [48]. Since the fornix is elongated as a rim in the direction towards the most anterior point of the crest, it may fulfil the task of stabilizing the tremendously enhanced surface of this crest like a T-girder in technical constructions.

Increased crests could allow the organisms to grow larger antennal muscles that are necessary for stronger antennal strokes [49, 50]. This is contradictory to recent research that found *Notonecta*-exposed *D*. *longicephala* swim more slowly [51]. In predation experiments, we often observe that *Notonecta* reorients captured prey *D*. *longicephala* until it can insert the proboscis into the ventral gap. In this regard, *Notonecta*-exposed *D*. *barbata* and *D*. *longicephala* probably share a common defensive feature, which is the narrowed ventral margin. In contrast, the unexposed shape actually could be exploited to direct the proboscis into the ventral gap.

Furthermore, prey capture seems to be complicated by the increased crest size. With a crest larger than the manipulating appendages of *Notonecta*, the grasp is weaker, and escape chances increase. This may be especially potent against smaller *Notonecta* instars. As previously observed, predation by various *Notonecta* instars affects *Daphnia* populations differently, i.e. through their choice of size [52]. To reach around the fully expressed crests, longer legs of the predator are necessary, which only later instars possess. Therefore, we hypothesize large *Notonecta* to be able to prey upon all *D*. *longicephala*, while smaller instars are dependent on smaller or unexposed specimens.

We anticipate that the different size classes of the two species reacting to *Notonecta*, *D*. *barbata* and *D*. *longicephala*, explain the partly contrasting morphological strategies. Furthermore, it is possible that small-sized *D*. *barbata* predominantly fall prey to juvenile *Notonecta* and are less attractive for later instars or even adult backswimmers in accordance with the theory of optimal foraging [53]. If that is the case, it may explain the varying morphologies in the two species. Maybe, the different alterations seen in *D*. *barbata* and *D*. *longicephala* are like excerpts of developing defensive traits as seen in *D*. *cucullata*, which counter various predators simultaneously in the different size classes of the ontogenetic development [24].

Generally, *Notonecta* prefers larger prey, suggesting a visual prey detection [54]. Nevertheless, *Notonecta* is thought to hunt using mechanoreception in addition to visual cues, as they are continuously active and also feed during the night [55, 56]. Therefore, apart from beneficial interactions of the *Notonecta*-exposed morphological features with the feeding apparatus, hydromechanical advantages for *D*. *longicephala* are suggested, allowing them to swim disguised [50].

#### *Chaoborus*-induced morphology

In the predator-prey system *Chaoborus-D*. *pulex*, we observed the prey to form a pronounced head region at the base of the neckteeth. We also noticed an increase in body depth, i.e. the dorso-ventral length was enlarged, as Tollrian already stated [23]. Neckteeth have been described in numerous publications [11, 12, 36, 57]. What has not been described so far, are the changes in lateral width observed here. In fact, the head capsule itself is comparatively thin, while the region of the neckteeth becomes significantly thicker. This is also mirrored in the central body, which is thickened in the predator exposed morph. Together with the increased tail spine length, these alterations corroborate the common hypothesis that neckteeth serve to outgrow the predator’s gape, and to impair the predator’s handling procedures, rendering *D*. *pulex* less graspable, independent of *Chaoborus’* strike efficiency [4, 5, 12, 23, 58]. Pastorok found that *Chaoborus* handled prey usually head first, suggesting an interaction of the neckteeth with the mouthparts, which could not be confirmed in a recent study [20, 59]. Therefore, we suggest the neckteeth may serve another purpose, e.g. altering the streamlining properties, as suggested for *D*. *longicephala*.

Also, the slimmed head morphology together with the increased thickness in the region around the heart may serve to improve stiffness as technically employed in cardboard or undulated surfaces of, e.g., tins [60]. In a previous publication [48], the model’s resolution was not adequate to reflect the undulating structure. With our results, we are now able to enhance the precision of such models.

Overall morphological alterations in *D*. *pulex* appear, in comparison to the other described species, rather minor. Nevertheless, they have repeatably been shown to correlate significantly with an increase of survival chances when encountering *Chaoborus* predation and therefore have a strong ecological effect [12, 61]. This underpins the hypothesis of a very ‘technical’ attack sequence, that can be disturbed with rather minor alterations of this ‘lock-and-key’-like system [5].

Furthermore, the altered morphology may not only complicate predator handling time, but also the detection, possibly by altered hydrodynamic trails [62]. The hypothesis of altered streamlining properties is especially supported by the finding that *Chaoborus*-exposed animals are also less frequently attacked [20].

#### *Gasterosteus*-induced morphologies

The morphological changes of *D*. *lumholtzi* are quite unique compared to the other defensive features in this study, as in this species we found the formation of a pointed helmet and an elongation of the tail spine, which was reported previously [15, 46, 63, 64]. Further, we were able to detect a previously unreported and potentially defensive structure similar to the pointed head and spine: the fornices on the sides of the head capsule are very pronounced in fish-exposed animals, extending the overall lateral width by up to 245 μm. This strategy to elongate structures orthogonal to each other was found in other *Daphnia* species (e.g. *Hyalodaphnia hypsicephala*, *D*. *magna*, *D*. *cucullata*, *Scapholeberis mucronata*) [65, 66].

Since small fish and especially juvenile stages (0+ juveniles) are generally regarded as gape-limited predators, it is possible that this cross-shaped body outline blocks the predator’s mouth and therefore significantly impedes fish suction feeding, even though the longitudinal axis is most relevant according to [67]. Even if a predator-exposed *D*. *lumholtzi* is being fully sucked into a fish’s mouth, there are still many interfering points e.g. the gills slits in which the prey item could get caught prior to full ingestion. Indeed, fish spit out and subsequently avoid induced *D*. *lumholtzi* more often (personal observation, [64, 68]).

Apart from this specific shape against fish predation, *D*. *lumholtzi*’s helmets function as a multitool also against *Chaoborus* predation, similar to the helmets of *D*. *cucullata* [24, 64].

We hypothesize the reduced lateral and dorso-ventral width of the carapace to be due to a trade-off between energetic and material costs. Furthermore, the shrinking in the dimension of the sagittal plane may allow the flow of water around the daphniids body, while the animal is wedged to the predator’s mouth by suction. Moreover, it is imaginable that drag forces are decreased by this shape alteration, reducing the forces pulling the *D*. *lumholtzi* into the fish’s mouth. Further, smaller bodies reduce visibility to fish [53, 69–71].

Apart from *D*. *barbata*, which forms much less pronounced helmets, *D*. *lumholtzi* is the only species in our study forming pointed helmets. In addition to this species, helmets are known as morphological feature in *D*. *cucullata*, *D*. *longispina* and *D*. *retrocurva*, just to name a few [16, 72, 73]. Whether similar three-dimensional alterations like fornices also occur in these and other species remains to be investigated.

### Procrustes distance as a measure of similarity

While morphological features are not necessarily detectable among species that share the same predator, Procrustes distance, especially average Procrustes distance, provides an objective measure of similarity.

The difference between the unexposed and predator-exposed morph of *D*. *pulex* is the smallest average partial Procrustes distance we measured. For the average full Procrustes distance, we determined values of 10.5 μm, i.e., the size has almost no influence on the altered form. Due to the calculation-order in Procrustes analyses, the full Procrustes distance is even slightly larger than the partial Procrustes distance here. This result is in accordance with the proposed anti-lock-and-key mechanism in this species [5], but is also influenced by the fact that *D*. *pulex* is the species in which we investigated the smallest individuals (Table 1). *D*. *barbata*, *D*. *lumholtzi* and *D*. *magna* have almost equally large intraspecific average full Procrustes distances (~30 μm), meaning that the pure shapes are altered with comparable effort in reaction to different predators. Nevertheless, as *D*. *magna* is obviously larger, the extent of alteration in comparison to body size in this species is lower (1% of body size) compared to *D*. *barbat*a and *D*. *lumholtzi* (2–3% of body size). *D*. *barbata* induced by *Triops* as well as *D*. *lumholtzi* show almost no alteration between partial and full Procrustes distance. Mostly shape, not size, is important in the morphological changes of these species. In contrast, the intraspecific average Procrustes distance of *D*. *magna* decreases from 80 μm in the partial analysis to 28 μm in the full Procrustes fit, rendering the size-effect obvious. *D*. *longicephala* has a strong shape as well as a size component in its alterations, as the partial average Procrustes distance is 261 μm and the full average Procrustes distance is 139 μm.

Between the species, smallest average partial distances occur in comparisons including *D*. *lumholtzi*. This species is potentially ‘ordinary’, i.e., has a combination of features found in the other species, like a roundish central body or an average sized and shaped head. Therefore, the average full Procrustes distance to the other species is comparatively small. Furthermore, both *D*. *barbata* morphs show a quite similar partial as well as full average Procrustes distance to *D*. *longicephala*. Not only the *Notonecta*-, but also the *Triops*-exposed morph of *D*. *barbata* has this small Procrustes distance to *D*. *longicephala*. Previous studies have shown that also *Triops*-defended morphs had a reduced mortality under predation by *Notonecta* [22], therefore the calculated Procrustes distances may indicate a shape similarity.

Moreover, the partial Procrustes distances measured for *D*. *magna* in comparison to all other species are quite large. As they decrease substantially for the full Procrustes distances, it becomes clear that size here explains differences to a major extent. Similar to *D*. *lumholtzi*, *D*. *magna*’s overall shape is not that different from the other species. For example, the average full Procrustes distance between *D*. *pulex* and *D*. *magna* is only 34.934 μm, a value similar to some intraspecific Procrustes distances.

Accordingly, Procrustes distance as a measure of difference is not able to predict predators, as the amount of alteration in form or shape is not specific for a predator. Nevertheless, Procrustes distance can indicate similar strategies, especially overall growth vs specific, local alteration. This can also give a hint on the costs of the potential defensive structures.

## Conclusions

Each encounter situation where the prey passes the predator undetected, increases prey survival chances. Likewise, each attack that is not followed by an instant ingestion provides the chance for the prey to escape. We showed that eco-responsive flexibility with the potential to enhance survival through multiple three-dimensional morphological alterations evolved. Based on them we were able to draw hypotheses on the actual function. A somewhat general strategy seems to be the formation of an elongated tail spine. In addition, we show previously unknown changes in the lateral dimension in all species investigated.

While some of the investigated *Daphnia* species seem to follow a similar strategy against one predator, others appear to use alternative tactics against the same threat. However, similar morphological features are also formed against different predators. Therefore, generalizations are limited. We speculate that different preferred body sizes potentially require different morphological adaptations. Our results therefore show that, despite their different size classes and evolutionary origins of *Daphnia* species, similar strategies evolved, which is in accordance with [35].

Apart from the above-mentioned results, average Procrustes distances can point to the degree and type of alteration and energetic investments that are necessary to form the altered morphologies. Accordingly, knowing the three-dimensional alterations is a cornerstone to answer not only functional questions but also considering inducible defenses’ trade-offs. For example, with our models, studies investigating the single mechanisms in depth, virtual simulations of the predatory attack or measurements of drag force alterations triggered by the observed three-dimensional alterations, just to name a few, can be undertaken.

## Supporting information

S1 Fig Body regions of *Daphnia*, illustrated with an undefended adult *Daphnia longicephala*. cc = central carapace, ce = compound eye, dm = dorsal margin, f = fornix, hc = head capsule, hr = heart region, r = rostrum, si = tail spine insertion, ts = tail spine, vm = ventral margin. Body directions are given in the coordinate system. ant = anterior, pos = posterior, dor = dorsal, ven = ventral, lat = lateral.
(TIF)

S1 File (DOCX)
