# Supplementary figures and images for: Thwarting predators? A three-dimensional perspective of morphological alterations in the freshwater crustacean Daphnia
Source: PLoS One. 2021 Jul 7;16(7):e0254263. doi: 10.1371/journal.pone.0254263 (PMC8263308; doi:10.1371/journal.pone.0254263)

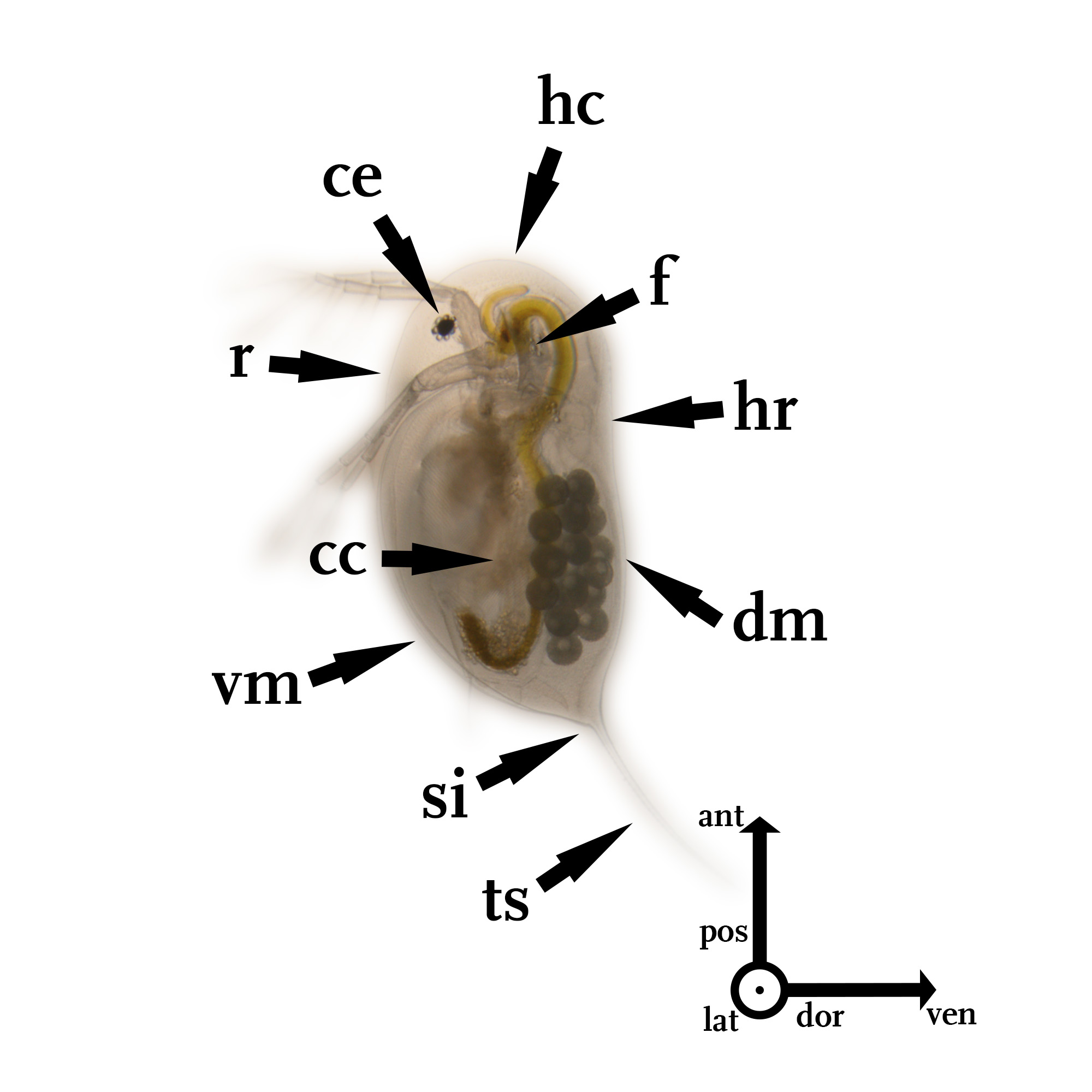

Supplement: S1 Fig — cc = central carapace, ce = compound eye, dm = dorsal margin, f = fornix, hc = head capsule, hr = heart region, r = rostrum, si = tail spine insertion, ts = tail spine, vm = ventral margin. Body directions are given in the coordinate system. ant = anterior, pos = posterior, dor = dorsal, ven = ventral, lat = lateral. (TIF) [file pone.0254263.s001.tif]
