# Supplementary material for: Thwarting predators? A three-dimensional perspective of morphological alterations in the freshwater crustacean Daphnia
Source: PLoS One. 2021 Jul 7;16(7):e0254263. doi: 10.1371/journal.pone.0254263 (PMC8263308; doi:10.1371/journal.pone.0254263)
Supplement: S1 File — (DOCX) [file pone.0254263.s002.docx]

**S1 Text. Detailed morphological alterations of *Triops*-exposed *Daphnia magna.***

*Triops*-exposed compared to unexposed *D. magna* (Fig 2A, B) have maximal alterations at the body margins as well as at the tail spine (>120 µm, displayed in shades of red in Fig 2C), which means the regions marked red are in total displaced by up to 120 µm compared to the unexposed morph, irrespective of any axis.

Deformations along the dorsoventral axis reveal that all vertices ventral of the body’s transversal axis are shifted ventrally (shades of blue in Fig 2D; negative x), while vertices dorsal of the axis are offset dorsally (shades of red in Fig 2D; positive x). The strength of the deformation increases with distance from the transversal axis (Fig 2D). On the head capsule points are strongly shifted dorsally (shades of red in Fig 2D; positive x). The location of the described vertices is significantly different between control and the defended animals (Fig 2E, p<0.05, q<0.01). These observations indicate that the animals become wider in dorso-ventral direction.

We observe a similar pattern in the plot for deformations in anterior-posterior direction: Vertices anterior of the body’s dorsoventral axis are shifted anteriorly (colored red in Fig 2F, positive y), vertices lying posterior are offset posteriorly (colored blue in Fig 2F, negative y). The translocations increase with distance to this axis (Fig 2F) is statistically significant (Fig 2G, p<0.05, q<0.01). These observations indicate an increase of body length in predator-exposed animals.

In lateral direction, we observed an increased body width centrally on the carapace (shades of red in Fig 2H, positive z), where a rim on the carapace is located. Furthermore, the lateral width of the fornix on the head capsule is increased, thereby vertices are moved in front of the drawing plane (shades of red in Fig 2H, positive z). At the same time, the posterior ventral margin and the dorsal margin are slightly slimmer in predator-exposed *D. magna*, moving them behind the drawing plane (shades of blue in Fig 2H, negative z). These are significant changes in the increments and reductions in lateral width (Fig 1I, p<0.05, q<0.01).

**S2 Text. Detailed morphological alterations of *Triops*-exposed *Daphnia barbata.***

An altered head capsule i.e. helmet is visible in the photographs (Fig 3A, B). Maximal changes of form occur at the tip of the tail spine and the head with shifts of >70 µm. Posterior regions of the ventral margin are altered with shifts ranging from 50-70 µm (Fig 3C; displayed in shades of red).

Shifts in dorsoventral direction show that, while the tail spine tip is mainly shifted dorsally (displayed in shades of red in Fig 3D), the ventral margin is translocated ventrally, making it more convex (displayed in shadows of blue in Fig 3D). These deformations are statistically significant (p<0.05 and q<0.01, Fig 3E). Apart from shifts in dorsoventral direction, the animals increase their body length, visible as shifts on the anterior (shades of red, Fig 3F) and posterior end (shades of blue, Fig 3F). These deformations show a statistical tendency (p<0.05 and q>0.01, Fig 3G). Furthermore, parts of the ventral margin are shifted in anterior direction (Fig 3F/G, central regions in shades of red).

In lateral dimension, the shape is asymmetric (Fig 3H, I); while the head is shifted to the animal’s right (Fig 3H, shades of blue at the head), the tail spine is displaced to the left (Fig 3H, shades of red at the tail spine). Additional statistically significant translocations are found at the carapace fold that is more pronounced in the lateral dimension in defended than in undefended *D. barbata* (Fig 3H, I, yellow-red coloring centrally on the animal). Not only this fold, but also the whole carapace is expanded by 15-20 µm in defended animals.

**S3 Text. Detailed morphological alterations of *Notonecta-*exposed *Daphnia barbata.***

Maximal changes between unexposed (Fig 4A) and *Notonecta*-exposed (Fig 4B) animals occur at the tail spine and at the tip of the helmet, the rostrum and the region between the head capsule and the head carapace (Fig 4C, shades of red).

The dorsal region around the heart is shifted ventrally (Fig 4D, shades of blue), while the rest of the body is either unaffected or shifted dorsally (Fig 4D, shades of red). These deformations are statistically significant (Fig 4E, p<0.05, q<0.01).

In anterior-posterior direction, the tip of the head and tail spine is more pointed compared to undefended animals. Overall, the posterior body parts are significantly shifted anteriorly (Fig 4F/G, shades of blue), except for parts of the tail spine and the region posterior to the head capsule-carapace transition (Fig 4F/G).

In lateral dimension, the width of the fold on the head and the tip of the helmet is increased. The carapace is not or just slightly thickened, at the center it is about 30-40 µm wider. The dorsal margin is also strengthened by 15-20 µm (Fig 4H, shades of red). Attenuation of lateral width is found on the tail spine, the ventral margin, and the ventral parts of the head capsule (Fig 4H, shades of blue). These deformations show a statistical tendency (p < 0.05 and q > 0.01, Fig 4I).

**S4 Text. Detailed morphological alterations of *Notonecta*-exposed *Daphnia longicephala.***

In comparison to unexposed *D. longicephala* (Fig 5A), the *Notonecta*-exposed morphotype is characterized by a pronounced crest (Fig 5B). The crest and the tail spine show the highest degree of overall deformation (>500 µm, Fig 5C, shades of red). Smaller overall shifts of up to 300 µm occur along the ventral margin of the carapace (Fig 5C, yellow-green).

Analyzing deformations along the dorso-ventral axis reveals that all vertices ventral of the body’s transversal axis are shifted ventrally (Fig 5D, shades of blue), while vertices dorsally appear to be offset dorsally (Fig 5D, shades of red). The magnitude of the deformation increases with distance to the axis. On the carapace, points are shifted towards the dorsal margin noticeably (Fig 5D, blueish zero alteration zone). The location of the described vertices is significantly different between control and the defended animals (p < 0.05 and q < 0.01, Fig 5E).

A similar pattern is visible in the plot for the anterior-posterior direction. Here, vertices located anterior of the dorsoventral axis connecting rostrum and the animal’s dorsal margin are shifted anterior (Fig 5F, shades of red). Vertices lying posterior are offset posteriorly (Fig 5F, shades of blue). Again, the translocations’ magnitude increases with distance to this axis. The described shifts are, as the other alterations, proved significant by the Wilcoxon-/FDR-analysis (p < 0.05 and q < 0.01, Fig 5G).

In lateral direction the folds on the head capsule are increased in thickness (>100 µm per body half), as well as the center of the carapace (>50 µm per body half) (Fig 5H, shades of red on the carapace and the head). The roundish region dorsally of the head fold is thinned by up to 100 µm (Fig 5H, per body half, shades of blue). Apart from these very strong alterations, the dorsal and ventral margins are decreased in thickness (Fig 5H -40 µm, shades of yellow-green). Furthermore, the most anterior parts of the head capsule show an increment of lateral thickness per body half of up to 50 µm (Fig 5H, shades of red). All above mentioned vertex translocations in lateral direction are proved significantly different by the Wilcoxon-/FDR-analysis (p < 0.05 and q < 0.01, Fig 5I).

**S5 Text. Detailed morphological alterations of *Chaoborus*-exposed *Daphnia pulex.***

Maximal deformation between unexposed and *Chaoborus*-exposed animals of *D. pulex* (Fig 6A/B) occurs in the neckteeth region (Fig 6C, >25 µm, shades of red).

This displacement is mainly due to a shift in dorsal direction (Fig 6D, shades of red). The described changes are proved significant (p<0.05 and q<0.01, Fig 6E).

Moreover, we observe a strong tendency of the neckteeth and parts of the central carapace region to be shifted towards the anterior end of the animal (Fig 6F, shades of red). These deformations show a statistical tendency but could not be proven significant (p<0.05 and q>0.01, Fig 6G).

In lateral dimension we observe a decrease in the head’s thickness (Fig 6H). Accordingly, also the fornix is attenuated. The heart region is characterized by an alternating pattern of positive and negative displacement in this direction. There is a strong tendency that the central carapace region becomes thicker by 5 to 10 µm (p<0.05, q>0.01, Fig 6I).

**S6 Text. Detailed morphological alterations of *Gasterosteus*-exposed *Daphnia lumholtzi.***

Obvious alterations of *D. lumholtzi* in *Gasterosteus* presence are an elongated tail spine and a pointed helmet (Fig 7A, B). The strongest alterations are observable at the tip of the tail spine and the tip of the pointed head capsule (Fig 7C, shades of red, >100 µm).

The induced helmet appears elongated ventrally (Fig 7D, shades of blue at the helmet). At the same time, the tail spine is offset dorsally (Fig 7D, shades of red at the tail spine tip). While the helmet is significantly altered (p<0.05, q<0.01, Fig 7E), the tail spine’s shift shows just a strong tendency (p<0.05, q>0.01, Fig 7E). In addition, the most anterior parts of the dorsal margin, adjacent of the rostrum, are shifted dorsally, thinning the animal, which is a significant alteration as well (Fig 7D/E, shades of red at the carapace, p<0.05, q<0.01).

The head is elongated anteriorly (Fig 7F, shades of red at the helmet), while the tail spine is shifted posteriorly (Fig 7F, shades of blue). Also, the posterior carapace area is shifted in anterior direction (Fig 7F, shades of green), indicating a shrinking of the central body. These changes are proven to be significant alterations as well (p<0.05, q<0.01, Fig 7G).

Strong lateral deformations occur at the fornices (Fig 7H insert). These laterally extruded body appendages of the head capsule enhance the absolute width of defended animals by 200 µm by being more pointed (100 µm per body half) (Fig 7H, shades of red). The rest of the body appears almost unaltered in lateral body width, and only small areas appear thinned in lateral dimension in defended animals, e.g. regions at the dorsal and ventral margins of the carapace (Fig 7H, shades of blue). The increased lateral width and the reduced width of the carapace are significant alterations (p<0.05, q<0.01, Fig 7I).
